# Development and validation of an anastomotic risk score for use in a randomized clinical trial on defunctioning stoma use in low anterior resection for rectal cancer

**DOI:** 10.1111/codi.70089

**Published:** 2025-04-10

**Authors:** Martin Rutegård, Ida Hed Myrberg, Caroline Nordenvall, Kalle Landerholm, Fredrik Jörgren, Peter Matthiessen, Jennifer Park, Josefin Segelman, Pamela Buchwald, Jenny Häggström

**Affiliations:** ^1^ Department of Diagnostics and Intervention, Surgery Umeå University Umeå Sweden; ^2^ Division of Clinical Epidemiology, Department of Medicine Solna Karolinska Institutet Stockholm Sweden; ^3^ Department of Molecular Medicine and Surgery Karolinska Institutet Stockholm Sweden; ^4^ Department of Pelvic Cancer Karolinska University Hospital Stockholm Sweden; ^5^ Department of Surgery Ryhov County Hospital Jönköping Sweden; ^6^ Department of Biomedical and Clinical Sciences Linköping University Linköping Sweden; ^7^ Department of Clinical Sciences Malmö Lund University Malmö Sweden; ^8^ Department of Surgery, Faculty of Medicine and Health Sciences Örebro University Örebro Sweden; ^9^ Department of Surgery, Sahlgrenska Academy Gothenburg University, Sahlgrenska University Hospital Gothenburg Sweden; ^10^ Department of Surgery Ersta Hospital Stockholm Sweden; ^11^ Department of Statistics, Umeå School of Business, Economics and Statistics Umeå University Umeå Sweden

**Keywords:** anastomosis, diverting stoma, insufficiency, leakage, prediction, total mesorectal excision

## Abstract

**Aim:**

The selective use of defunctioning stomas in anterior resection for rectal cancer hinges on accurately predicting anastomotic leakage. The aim of this study was to develop a prediction model for use in a prospective randomized clinical trial.

**Method:**

Colorectal Cancer Database (CRCBaSe) Sweden was used to identify patients who underwent low anterior resection for rectal cancer 2007–2021. Eligibility criteria mirrored the forthcoming SELective defunctioning Stoma Approach in low anterior resection for rectal cancer (SELSA) trial, including patients <80 years of age and with American Society of Anaesthesiologists' (ASA) physical status grade of <III; further, patients without a defunctioning stoma were excluded. The outcome comprised anastomotic leakage within 30 days or in‐hospital. Candidate predictors included age, sex, ASA grade, cardiovascular disease, diabetes, body mass index (BMI), tumour stage, tumour height, and neoadjuvant therapy. Seven models were developed and internally validated using bootstrapping. A threshold of a predicted leakage risk of ≤10% was chosen for trial implementation. Validation was conducted using chart‐reviewed data from a nested cohort.

**Results:**

Of the 2727 eligible patients, 199 (7.3%) were registered with an anastomotic leakage. All models demonstrated similar performance, with prediction instability observed for risks exceeding 12.5%. The preferred model included three significant predictors: male sex (OR 2.00; 95% CI: 1.45–2.75), BMI >30 kg/m^2^ (OR 1.82; 95% CI: 1.21–2.74), and radiotherapy (OR 1.90; 95% CI: 1.35–2.69). The bootstrapped area under the curve (AUC) was 0.64 (95% CI: 0.62–0.65), with a negative predictive value of 94.6% (95% CI: 93.7%–95.6%). For the validation cohort, the corresponding estimates were 0.66 (95% CI: 0.59–0.74) and 89.5% (95% CI: 86.2%–92.5%).

**Conclusion:**

Accuracy of anastomotic leakage prediction using registry‐based data is moderate; however, the model's ability to rule out a >10% risk is considered appropriate for trial use.

**What does this paper add to the literature?**

Prediction of anastomotic leakage after low anterior resection for rectal cancer is crucial for judicious use of defunctioning stomas. A model developed using registry‐based and validated with chart‐reviewed data rendered an acceptable model for trial use, comprising male sex, obesity, and neoadjuvant radiotherapy.

## INTRODUCTION

Anastomotic leakage after low anterior resection for rectal cancer poses a clinical dilemma. Multicentre cohort studies describe leak rates ranging from 20% to 24% [1, 2], a cause of morbidity [3] as well as mortality [4]. Defunctioning stomas are in widespread use to reduce the risk of having or at least mitigate the consequences of a leak, but are accompanied with their own set of problems such as dehydration, kidney injury [5] and the risk of non‐reversal [6]. Efforts have been made to investigate a more selective approach to defunction patients undergoing low anterior resection, with at least one randomized trial currently recruiting [7]. Our research group is planning a similar study, the SELective defunctioning Stoma Approach in low anterior resection for rectal cancer (SELSA) trial [8].

The selection process in the SELSA trial hinges upon the potential to identify patients with a perceived lower risk of anastomotic leakage, for whom omittance of a defunctioning stoma will be deemed acceptable. Established risk factors for leakage after low anterior resection include male sex, radiotherapy, obesity, active smoking, diabetes and other comorbidities, multiple stapler firings in minimally invasive surgery, and intraoperative bleeding [9, 10, 11, 12, 13]. Of these, the SELSA trial will exclude patients with active smoking, excessive bleeding and multiple stapler firings.

The aim of the present study was to develop and validate a risk score for anastomotic leakage after low anterior resection for rectal cancer, specifically for use in the SELSA trial, based on well‐known causes of leakage.

## METHOD

### Checklist for the reporting of prediction studies

This article was written in accordance with the Transparent Reporting of a multivariable prediction model for Individual Prognosis Or Diagnosis (TRIPOD) checklist for the reporting of prediction studies [14].

### Data source

#### Registry data

A nationwide registry study was performed based on *Colorectal Cancer Base Sweden* (*CRCBaSe*), a registry‐linkage originating from a national quality register, *the Swedish Colorectal Cancer Registry* (*SCRCR*), containing nearly all Swedish patients diagnosed with colorectal cancer, starting in 1995 for rectal cancer and 2007 for colon cancer. Compliance has been assessed at 98.8% for rectal cancer [15], universally defined in Sweden as an adenocarcinoma with its inferior margin within 15 cm from the anal verge as measured with rigid sigmoidoscopy. Data are prospectively registered during treatment and follow‐up. Patient and tumour characteristics such as age, sex, Body Mass Index (BMI), American Society of Anaesthesiologists' (ASA) physical status grade, tumour location and tumour stage are reported in detail, as well as preoperative treatment and peri‐ and postoperative data. Several other national registries are incorporated into CRCBaSe. *The Prescribed Drug Registry* records information on all dispensed prescription drugs since 2005 [16]. This registry contains information on Anatomical Therapeutic Chemical (ATC) code, dosage and date of prescription as well as dispensations. *The National Patient Registry* includes dates and codes of diagnoses and surgical procedures. Data on inpatient care has been collected since 1964 (with nationwide coverage since 1987) and outpatient data since 2001, with a national coverage of more than 99% [17]. The *Swedish Cancer Registry* was established in 1958 and contains all primary cancers diagnosed in Sweden, with reporting mandated by law; the coverage of this registry is nearly complete (98%) [18].

#### Validation cohort

Patients operated with low anterior resection (with total mesorectal excision) for rectal cancer during 2014–2018 at 11 centres in Sweden were identified using the SCRCR and theatre lists, comprising the so‐called RectoLeak study cohort [2]. Registry data were supplemented with detailed chart‐reviewed information, including intraoperative data such as type of mesorectal excision and intraoperative complications, as well as comprehensive categorization of anastomotic leakage based on the consensus definition provided by the International Study Group of Rectal Cancer [19]. These patients were thus nested into the development data set. However, any additional information obtained from reviewing their charts was used only in the validation phase and not during model development.

### Study design

#### Eligibility

Eligible patients, operated with anterior resection for rectal cancer in 2007–2021, were identified in the SCRCR. As there are no registry data on the extent of mesorectal excision, patients with a tumour above 12 cm were excluded, assuming that tumours with an inferior margin of 12 cm and below were operated with total mesorectal excision down to the pelvic floor. The other eligibility criteria are described in Table 1, essentially depicting reasons for exclusion from the planned SELSA trial. These include previous rectal cancer or synchronous colorectal cancer, previous pelvic irradiation, emergency surgery, excessive intraoperative blood loss, intraoperative bowel perforation, and beyond total mesorectal excision surgery. As the SELSA trial aims to randomize within a low‐risk population in terms of leak‐induced mortality, patients aged ≥80 years and with an ASA physical status grade of ≥III were also excluded. Of particular importance is the additional exclusion criterion of no defunctioning stoma use; this is applied in this prediction study only, for several reasons: (i) the tested intervention in the SELSA trial comprises stoma use; (ii) the vast majority of patients operated with low anterior resection during the study period were defunctioned [2] and (iii) defunctioning stomas might delay rather than prevent leakage [1, 2]. Therefore, this prediction study excludes non‐defunctioned patients and defunctioning stoma is not included as a candidate predictor.

**TABLE 1:** Eligibility criteria for the prediction study aligned with the planned SELSA trial (except for the planned intervention of no defunctioning stoma).

Eligibility criteria
*Inclusion criteria*
All patients operated 2007–2021
Tumour location in the rectum
Anterior resection performed
Tumour height ≤ 12 cm from the anal verge
*Exclusion criteria*
Emergency surgery
Age ≥ 80 years
ASA grade ≥ III
Previous pelvic irradiation^a^
Previous rectal cancer
Synchronous colorectal cancer
Permanent stoma operation
Beyond TME surgery
Blood loss ≥500 mL if open or converted surgery
Blood loss ≥250 mL if laparoscopic surgery
Intraoperative bowel perforation

*Note*: No defunctioning stoma.

Abbreviations: ASA, American Society of Anesthesiologists; TME, total mesorectal excision.

^a^ Using codes from the Patient Registry: men: Z510 AND C61; women: Z510 AND (C51 OR C52 OR C53 OR C54); both sexes: Z510 AND C67.

#### Predictors

This study included candidate predictors based on availability as well as previous literature, indicating a potential role in the development or prediction of anastomotic leakage. These comprised age, sex, ASA grade, clinical local tumour extent (cT), clinical nodal stage (cN), receipt of neoadjuvant radiotherapy, BMI, prevalent diabetes mellitus, manifest cardiovascular disease and tumour height. Of note, patients registered as having received chemotherapy only were included in the chemoradiotherapy group.

Prevalent diabetes mellitus was derived using data from the *National Patient* and *Prescribed Drugs Registries* (full definition in Table S1). Manifest cardiovascular disease was derived using diagnostic and procedural codes from the *National Patient Registry*, reflecting previous events such as acute myocardial infarction, percutaneous and operative interventions for atherosclerotic disease, and so on (full definition in Table S2).

#### Outcome

The outcome was anastomotic leakage within 30 days or during the index admission, regardless of day of diagnosis. The registry does not provide a formal definition of leakage. The variable has been evaluated for rectal cancer surgery and found to be underreported (29%) when compared to an international consensus definition [17], whereas almost no false positives could be found [18]. Moreover, the registry does not include date and modality of detection for anastomotic leakage.

### Statistical analysis

All statistical analyses were performed using R 4.3.2 statistical software (R, 2023) [20].

#### Sample size calculation

The work on sample size calculations for prediction models in binary outcomes by Riley et al. [21] was applied. For this calculation, the largest model with 14 parameters was used. The anticipated Cox–Snell *R* ^2^ was set at 0.05 (corresponding to an area under the curve [AUC] of 0.726), the anticipated outcome proportion at 0.08 (expected anastomotic leak rate) and the shrinkage factor at 0.9 (recommended to decrease overfitting). These assumptions rendered a minimum required sample size of 2450 observations with 196 events, and a 14 events per candidate predictor parameter.

#### Missing data

Multiple imputation with chained equations was used to impute missing data for BMI, cN, cT and neoadjuvant therapy, with five imputations generated [22]. The imputation model included the outcome variable anastomotic leakage (binary) as well as all 10 predictors: age (continuous), sex (binary), ASA (binary), cT (4 categories), cN (3 categories), neoadjuvant radiotherapy (3 categories), BMI (continuous), diabetes (binary), cardiovascular disease (binary) and tumour height (continuous). The imputation was made in RStudio using the mice package [23].

#### Model development

Multivariable prediction models were fitted in each imputed dataset using a logistic regression model. Model coefficients were estimated for each imputed dataset, and predictions based on these were made, before being combined across imputations using Rubin's rules [24]. Predictor effects in the models are reported as odds ratios with 95% confidence intervals. Analyses were undertaken using the glm function in RStudio. Automated variable selection methods were not used, since all variables were predetermined based on the literature and expert opinion. To ensure model parsimony, variables believed to have weak influence à priori and that showed minimal or no association in multivariable analysis were excluded in later models. In addition, interactions between the predictors were not considered to maintain simplicity. The predictors age (years; continuous), sex (binary: male, or female), ASA grade (binary: I or II), cT (categorical: 1–2, 3, 4, or X: undefined), cN (categorical: 0, 1–2, or X: undefined), diabetes (binary: no, or yes) and cardiovascular disease (binary: no, or yes) were all used with the same forms throughout. BMI was treated as either a continuous variable or as a binary variable (≤30 kg/m^2^, >30 kg/m^2^). Neoadjuvant therapy was either used with three categories (none, radiotherapy, or chemoradiotherapy), or as a binary variable (none, any radiotherapy). Tumour height was treated as a continuous variable or with three categories (≤8, 9–10, or 11–12 cm). Table S3 provides an overview of the evaluated models with included predictors. In the case of BMI and tumour height, their categorical versions were derived from the imputed continuous variables. The linearity assumption was visually assessed by plotting the logit of the model predictions against the values of the continuous predictors and comparing the linear fit with a smoothed loess fit.

#### Internal validation using bootstrapping

Internal validation was conducted using bootstrapping with 500 samples, with each bootstrap sample consisting of *n* = 2727 observations sampled with replacement from the model development population [25]. The imputation and model development steps were performed in each of the generated bootstrap samples. The predictive performance of each model was evaluated within the respective bootstrap sample as well as in the original dataset. Model stability was assessed through probability distribution and calibration instability plots [26]. Performance metrics included calibration and discrimination. Calibration was quantified using the calibration slope and intercept (calibration‐in‐the‐large) with calibration plots displaying the agreement between predicted and observed outcome probabilities through smoothed (loess) calibration curves. Discrimination was assessed using the AUC c‐statistic.

#### Classification using a determined threshold

Based upon clinical judgement, a predicted risk of anastomotic leakage >10% using registry data was deemed too high to render eligibility for randomization in the future trial. This decision considered potential underreporting and the incidence of late leakage, not captured in the registry data [2]. The observed outcome and pooled predictions from all models developed based on the original data were used to classify patients using this threshold, providing sensitivity, specificity and negative predictive values. This was later used to inform the preferred model choice.

#### Scoring system development

To facilitate usability, regression coefficients from each developed model were used to construct corresponding scoring systems [27, 28]. Points for each predictor category were derived by dividing “regression units” by the maximum regression coefficient in the model, then rounding to the nearest integer. To enable scoring, continuous predictors were categorized. Dividing by the maximum regression coefficient ensured simple scoring systems with low maximum point totals.

#### Application in validation cohort

To validate the findings in the development data, the models were applied to the RectoLeak study cohort [2]. The same eligibility criteria outlined in Table 1 were used in the same manner to select appropriate patients, with the exception that the tumour height criterion was replaced with total mesorectal excision (thus excluding patients operated with partial mesorectal excision).

## RESULTS

### Study participants

**FIGURE 1:**
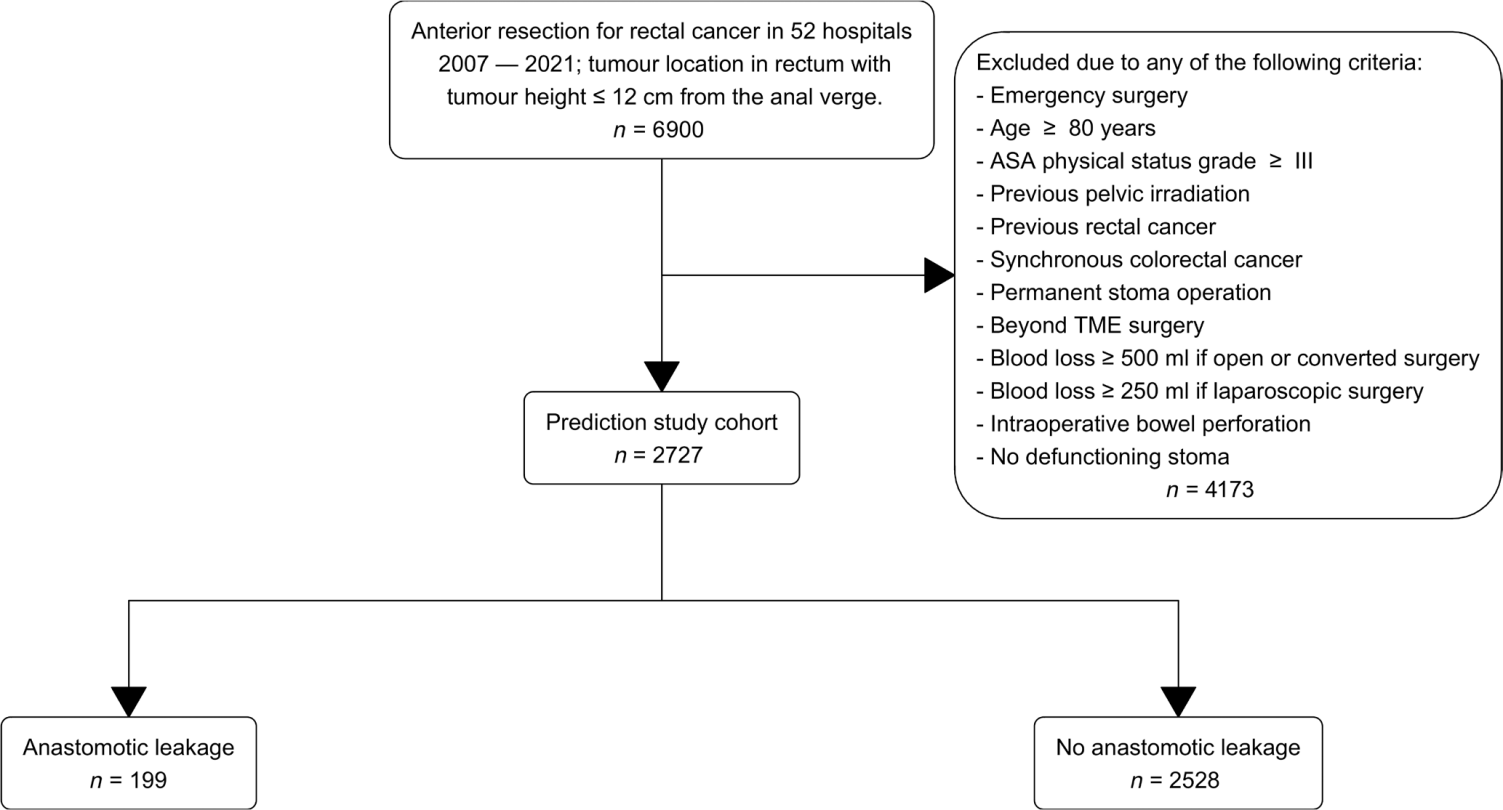
Prediction study flow chart.

The model development data consisted of eligible patients from 52 hospitals across Sweden. Of the 2727 patients available for model development, 199 (7.3%) were registered with an anastomotic leakage (Figure 1). In total, there were 3.3% incomplete observations. Characteristics for the model development cohort are given in Table 2.

**TABLE 2:** Baseline characteristics of the 2727 patients included in the SELSA prediction cohort, operated with anterior resection for rectal cancer in Sweden 2007–2021, stratified by presence of anastomotic leakage and derived from sample including missing values.

	No leak (*N* = 2528)	Leak (*N* = 199)	Overall (*N* = 2727)
Age (years)	65 (57; 71)	63 (57; 70)	65 (57; 71)
*Sex*			
Female	1167 (46.2%)	59 (29.6%)	1266 (45.0%)
Male	1361 (53.8%)	140 (70.4%)	1501 (55.0%)
*Body mass index* (*kg/m* ^*2*^)			
<30	2193 (86.7%)	164 (82.4%)	2357 (86.4%)
≥30	261 (10.3%)	32 (16.1%)	293 (10.7%)
Missing	74 (2.9%)	3 (1.5%)	77 (2.8%)
*ASA physical status grade*			
I	791 (31.3%)	71 (35.7%)	862 (31.6%)
II	1737 (68.7%)	128 (64.3%)	1865 (68.4%)
*Diabetes*			
No	2351 (93.0%)	178 (89.4%)	2529 (92.7%)
Yes	177 (7.0%)	21 (10.6%)	198 (7.3%)
*Manifest cardiovascular disease*			
No	2234 (88.4%)	168 (84.4%)	2402 (88.1%)
Yes	294 (11.6%)	31 (15.6%)	325 (11.9%)
Tumour height (cm)	9 (8; 11)	9 (8; 10)	9 (8; 11)
*Clinical T stage*			
cT1‐2	772 (30.5%)	43 (21.6%)	815 (29.9%)
cT3	1469 (58.1%)	141 (70.9%)	1610 (59.0%)
cT4	188 (7.4%)	9 (4.5%)	197 (7.2%)
cTX	86 (3.4%)	6 (3.0%)	92 (3.4%)
Missing	13 (0.5%)	0 (0.0%)	13 (0.5%)
*Clinical N stage*			
cN0	1093 (43.2%)	76 (38.2%)	1169 (42.9%)
cN1‐2	1320 (52.6%)	115 (57.8%)	1445 (53.0%)
cNX	99 (3.9%)	7 (3.5%)	106 (3.9%)
Missing	6 (0.2%)	1 (0.5%)	7 (0.3%)
*Clinical M stage*			
cM0	2388 (94.5%)	185 (93.0%)	2573 (94.4%)
cM1	121 (4.8%)	9 (4.5%)	130 (4.8%)
cMX	14 (0.6%)	4 (2.0%)	18 (0.7%)
Missing	5 (0.2%)	1 (0.5%)	6 (0.2%)
*Neoadjuvant radiotherapy*			
None	904 (35.8%)	45 (22.6%)	949 (34.8%)
Radiotherapy only	1142 (45.2%)	115 (57.8%)	1257 (46.1%)
Chemoradiotherapy^a^	481 (19.0%)	39 (19.6%)	520 (19.1%)
Missing	1 (0.0%)	0 (0.0%)	1 (0.0%)
*Surgical approach*			
Open	1396 (55.2%)	101 (50.8%)	1497 (54.9%)
Minimally invasive	1068 (42.2%)	96 (48.2%)	1164 (42.7%)
Converted to open	64 (2.5%)	2 (1.0%)	66 (2.4%)
Intraoperative bleeding (mL)	100.0 (50.0; 250.0)	150.0 (62.5; 250.0)	100.0 (50.0; 250.0)

*Note*: Continuous variables are presented with median and interquartile range; categorical variables are shown in number and column percent.

Abbreviation: ASA, American Society of Anesthesiologists.

^a^ 20 out of 520 were registered as having had chemotherapy only.

### Model development and performance

A total of seven prediction models were developed and evaluated. No major deviations from linearity were found for the models containing continuous predictors. The largest model consisted of ten predictors, where there was little evidence of an association with the risk of anastomotic leakage for half of the predictors in multivariable analysis. Male sex, increasing BMI and neoadjuvant therapy were predictors clearly associated with an increased risk of leakage in all models (Tables S4 and S5).

**FIGURE 2:**
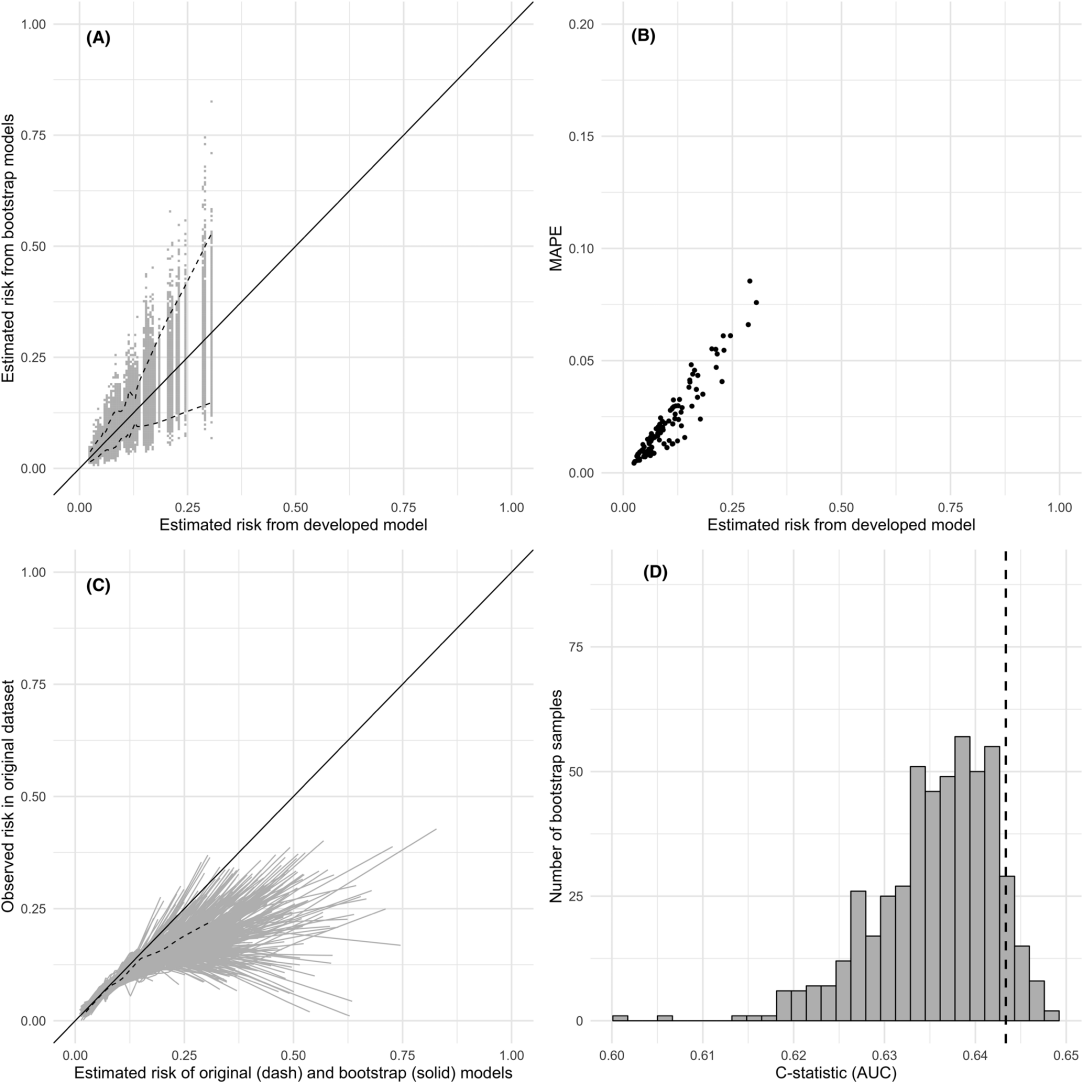
Model 5 instability plots. (A) Prediction instability plot. Plot of (pooled) predictions based on original data (x‐axis) versus (pooled) predictions based on bootstrap samples. (B) Mean absolute prediction error (MAPE) instability plot. Plot of (pooled) predictions based on original data versus MAPE in the bootstrap samples. (C) Calibration instability plot. Plot of calibration curves based on bootstrap samples (grey) and original data (black dashed). (D) Bootstrap test performance. Histogram of area under the curve (AUCs) based on bootstrap samples (5 imputations) used to predict outcome in the original five imputed datasets.

Instability plots (Figure 2 and Figures S1–S6) showed high variability in individual‐level predictions and calibration curves, implying an unstable model in the development population. Apparent performance in the development dataset and bootstrap test performance are reported in Table S6, Figure 2 and Figures S1–S6. For instance, AUCs for Model 6 were 0.64 (95% CI: 0.60–0.68) in the development dataset, and 0.64 (95% CI: 0.62–0.64) in the bootstrap test data. Apparent calibration in the model development data was satisfactory for predicted risks up to 10%, with the calibration curve closely following the diagonal line of ideal calibration (where predicted risks exactly match observed outcomes, see Figure S5). Model 6 resulted in bootstrap test calibration slope closest to unity, 0.85 (95% CI: 0.65–1.14), with a corresponding bootstrap test calibration intercept (calibration in the large) of −0.11 (95% CI: −0.26–0.06) suggesting poor calibration for some patients and overall over‐prediction of risks. All models yielded similar results (see Table S6, Figure 2 and Figures S1–S6). Over‐prediction was evident in 10%–20% of the population with a predicted risk higher than 12.5% as indicated by most bootstrap calibration curves lying below the diagonal on the calibration plots in this range (where predicted risks exceed observed outcomes).

### Classification and application

Next, the developed models were used to classify the rectal cancer cohort into a predicted risk of leakage >10% or not. All models performed similarly, with a negative predictive value (defined as the proportion of patients predicted to have no leakage who indeed experienced no observed leakage) close to 0.95 in all models (Table S7).

A scoring system for each model is presented in Table S8. The scoring systems maximum point totals ranged from 3 to 6. For all scoring systems, point totals 0 and 1 correspond to predicted risks (based on the developed logistic models) below the preferred threshold of 10% leakage risk. The distributions of predicted risks within different scoring strata can be seen in Table S9. For details on the derivation of the scoring systems, we refer to Sullivan et al. [28] and the results provided in Tables S10–S16.

### Model development synthesis

Based on ease of use, being a parsimonious model, and not excluding too many patients in the forthcoming trial, Model 5 was chosen as the preferred model. This model was also the least overfitted, while all models produced similar optimism corrected AUCs. The characteristics of this model are summarized in Table 3 and instability plots are provided in Figure 2.

**TABLE 3:** Summary of preferred prediction model.

Characteristic	Estimate (95% CI)
*Performance*	
Original train AUC	0.644 (0.606, 0.683)
Bootstrap test AUC	0.636 (0.621, 0.645)
Optimism corrected AUC	0.629 (0.590, 0.668)
*Calibration*	
Original train calibration slope	0.907 (0.645, 1.169)
Bootstrap test calibration slope	0.809 (0.621, 1.049)
Original train calibration intercept	−0.106 (−0.252, 0.040)
Bootstrap test calibration intercept	−0.109 (−0.267, 0.059)
*Significant ORs*	
Male sex	2.00 (1.45, 2.75)
BMI >30 kg/m^2^	1.82 (1.21, 2.74)
Neoadjuvant therapy	1.90 (1.35, 2.69)
*Classification in development cohort*	
Sensitivity	50.8 (24.8, 64.6)
Specificity	67.8 (59.6, 87.9)
Negative predictive value	94.6 (93.7, 95.6)
Positive predictive value	11.1 (10.1, 14.3)
*Classification using scoring system in development cohort*	
Sensitivity	62.8 (9.0, 98.4)
Specificity	59.6 (8.4, 95.2)
Negative predictive value	95.3 (93.0, 98.6)
Positive predictive value	10.9 (7.1, 13.9)
*Validation cohort characteristics*	
AUC	0.665 (0.589, 0.741)
Sensitivity	31.3 (18.8, 44.6)
Specificity	82.7 (79.1, 86.5)
Negative predictive value	89.5 (86.2, 92.5)
Positive predictive value	20.3 (11.9, 30.2)

Abbreviations: AUC, area under the curve; BMI, body mass index; CI, confidence interval; OR, odds ratio.

This model has an optimism corrected AUC of 0.63 (95% CI: 0.59–0.67), bootstrap calibration slope of 0.81 (95% CI: 0.62–1.05) and classified 33.5% of the cohort with a predicted anastomotic leakage risk of more than 10%. The significant predictors in this model were male sex (OR 2.00; 95% CI: 1.45–2.75), BMI >30 kg/m^2^ (OR 1.82; 95% CI: 1.21–2.74), and neoadjuvant radiotherapy (OR 1.90; 95% CI: 1.35–2.69). The scoring system for this model rendered one point each for male sex, high BMI, and neoadjuvant therapy, where a summative score of 0–1 indicated a predicted leak risk ≤10%, constituting 58.0% of the cohort (Tables S8 and S9).

### Validation cohort findings

Some 398 patients met the eligibility criteria, as outlined in Table 1, except for the tumour height criterion being replaced with total mesorectal excision. After excluding nine patients due to missing data on predictors or outcome, 389 patients remained for validation. Of these, 48 (12.3%) had chart‐reviewed anastomotic leakage within 30 days of surgery.

Using the chosen model to predict anastomotic leakage risk >10% in the validation cohort rendered an AUC of 0.66 (95% CI: 0.59–0.74). Sensitivity was 31.3% (95% CI: 18.8%–44.6%), specificity was 82.7% (95% CI: 79.1%–86.5%), and the negative predictive value was 89.5% (95% CI: 86.2%–92.5%).

## DISCUSSION

In this registry‐based prediction study, we developed several prediction models for anastomotic leakage following low anterior resection for rectal cancer, using candidate predictors well‐supported by the literature. A parsimonious model was chosen and tested on a chart‐reviewed validation cohort, which provided more detailed information on anastomotic leakage yielding acceptable results. The final prediction model identified male sex, obesity, and neoadjuvant therapy as similarly equally important risk factors, and as such will be utilized in the upcoming SELSA trial.

Albeit calibration was good in the low‐risk range, the primary focus of both the current study and the future SELSA trial, the chosen model rendered only a moderate AUC. Nevertheless, this is in line with the current literature on prediction of anastomotic leakage [29]. It is notoriously difficult to predict such leakage, and apparent limitations in the present registry‐based study include not having detailed patient data on, for example, preoperative inflammatory state [30, 31], current smoking and alcohol use [12] as well as malnutrition [32]. In the development data, we also had to assume that total mesorectal excision was performed based on tumour height, which probably introduced some misclassification. Another important limitation was the necessity to include only defunctioned patients, as these constituted most of the source cohort; to include also non‐defunctioned patients would have imparted a risk of considerable bias, as this minority in all likelihood consists of highly selected patients. However, there are known problems when assessing anastomotic leakage in defunctioned patients, not the least underreporting [33] and delays in diagnosis [2]. The derived prediction model performed similarly when applied to a cohort with more detailed data, although the negative predictive value was slightly lower, likely due to the higher incidence of anastomotic leakage in the chart‐reviewed cohort. The advantages of using a national registry‐based cohort are the sample size, systematic data collection and generalizability of the results. Unlike most prediction studies that often have small sample sizes, this study's large cohort allowed for reasonably accurate estimations. To ensure high‐quality analysis, a thorough understanding of the included registries and variables was essential. This expertise was ensured among the authors, and only carefully selected variables were incorporated into the study. The developed scoring system, despite some oversimplification, reflects the underlying model and should be straightforward to use.

The findings from this prediction study cannot yet be recommended for wider clinical use before proper external validation has been undertaken. The current validation involved evaluating the model on a cohort nested within the development set, with some overlap in the study years. External validation should ideally include other countries with similar healthcare systems and should be conducted prospectively. However, we developed a prediction model and scoring system designed to support patient selection for randomization in the planned SELSA trial, also providing an opportunity for validation.

While the chosen model included known and established predictors for anastomotic leakage, diabetes and cardiovascular disease did not emerge as strong predictors in the present study. This contrasts with the findings in a recent meta‐analysis on diabetes [9], whereas the association between cardiovascular disease and leakage remains less clear [34]. Several factors could explain this discrepancy, including misclassification in registry data, which may lead to underestimation of potential associations, but also that this cohort was limited to ASA grade I–II patients, likely excluding patients with severe systemic disease. Importantly, the lack of association with diabetes contradicts the findings of the GRECCAR‐17 trial's prediction model [7], which was developed using transanal total mesorectal excision registry data [35]. This difference might reflect variations in patient selection, or the impact of effective diabetes treatment with appropriate glycaemic control in the present study, mitigating anastomotic leakage risks.

It is essential to develop algorithms to predict the risk of anastomotic leakage after low anterior resection. For the algorithm to be clinically useful, it must be straightforward to implement in daily practice. Using a national registry‐based approach, male sex, obesity, and neoadjuvant radiotherapy were identified as key predictors, each contributing one risk point. This simple scoring system can be easily integrated into clinical settings; however, further validation is required to confirm its robustness. Future predictive studies with both larger sample sizes and more detailed data are certainly necessary.

## AUTHOR CONTRIBUTIONS

**Martin Rutegård:** Conceptualization; investigation; funding acquisition; writing – original draft; methodology; writing – review and editing; validation; project administration; data curation; resources. **Ida Hed Myrberg:** Writing – review and editing; validation; software; project administration; data curation; resources; methodology; supervision. **Caroline Nordenvall:** Conceptualization; funding acquisition; writing – review and editing; project administration; resources; supervision. **Kalle Landerholm:** Conceptualization; investigation; writing – review and editing. **Fredrik Jörgren:** Writing – review and editing; investigation; conceptualization. **Peter Matthiessen:** Conceptualization; investigation; writing – review and editing; validation. **Jennifer Park:** Conceptualization; investigation; writing – review and editing; validation. **Josefin Segelman:** Conceptualization; investigation; validation; writing – review and editing. **Pamela Buchwald:** Conceptualization; investigation; funding acquisition; writing – review and editing. **Jenny Häggström:** Conceptualization; investigation; writing – original draft; writing – review and editing; methodology; validation; visualization; software; formal analysis; data curation; supervision.

## FUNDING INFORMATION

Region Västerbotten (RV‐991591), Swedish Cancer Society (23 3056 Fk, 23 3221S), the Swedish Cancer and Allergy Foundation, the Stockholm Cancer Society, Swedish Research Council (VR 2023‐06400).

## CONFLICT OF INTEREST STATEMENT

None declared.

## ETHICS STATEMENT

The study was approved by the Regional Board of the Ethical Committee in Stockholm, Sweden (DNR: 2014/71‐31, 2018/328‐32, 2021‐00342, 2023‐03305‐02).

## Supporting information


Data S1

## Data Availability

The data that support the findings of this study are available from Colorectal Cancer DataBase Sweden (CRCBaSe) steering group. Restrictions apply to the availability of these data, which were used under license for this study. Data are available from the author(s) with the permission of Colorectal Cancer DataBase Sweden (CRCBaSe) steering group.
