# Supplementary material for: Development and validation of an anastomotic risk score for use in a randomized clinical trial on defunctioning stoma use in low anterior resection for rectal cancer
Source: Colorectal Dis. 2025 Apr 10;27(4):e70089. doi: 10.1111/codi.70089 (PMC11986403; doi:10.1111/codi.70089)
Supplement: Supplementary file 1 — Data S1 [file CODI-27-0-s001.docx]

**Supplementary Table 1.** Definition of diabetes mellitus.

| Prevalent diabetes mellitus before surgery was defined as fulfilling at least one of the following criteria before surgery: |
| --- |
| - A minimum of two medical records of diabetes diagnosis in the National Patient Registry (ICD-10 codes E10-E14 and ICD-8/9 code 250) |
| - One medical record of diabetes diagnosis in the National Patient Registry and one dispensed prescription for any antidiabetic drug (ATC-code A10) in the Prescribed Drugs Registry |
| - A minimum of two dispensed prescription of antidiabetic drugs in the Prescribed Drugs Registry during the last year before surgery |

**Supplementary Table 2.** Definition of manifest cardiovascular disease. ICD codes were derived from the National Patient Registry. Any type or code listed below registered before surgery was sufficient to fulfil the definition.

| Type of cardiovascular disease/intervention | ICD codes |
| --- | --- |
| Coronary atherosclerosis | I11, I13, I20-I25, I44-I50, I60-I69 (excluding I67.1, I67.5, I67.7, I68), I70-I73 |
| Acute myocardial infarction | I21, I22 |
| Procedures on coronary arteries | FN, FCA60, FCA70, FCA80 |
| Revascularisation of the thoracic aorta | VAH, VAJ, VAN, VAQ |
| Revascularisation of the abdominal aorta | VBF, VBJ, VBN, VBP, VBQ |
| Revascularisation of the brachiocephalic trunk | VCF, VCJ, VCN, VCP, VCQ |
| Endarterectomy of the subclavian artery | VEF |
| Revascularisation of mesenteric arteries | VGF, VGJ, VGN, VGP, VGQ |
| Revascularisation of the iliac artery | VHF, VHJ, VHN, VHP, VHQ |
| Revascularisation of the femoral/popliteal artery | VJE, VJJ, VJN, VJP, VJQ |
| Revascularisation of infrapopliteal arteries | VLF, VLJ, VLN, VLP, VLQ |

**Supplementary Table 3.** Model overview.

| Model | Predictors (forms) |
| --- | --- |
| 1 | Age (continuous), sex (binary), ASA grade (binary), cT (4 categories), cN (3 categories), neoadjuvant therapy (3 categories), BMI (continuous), diabetes (binary), cardiovascular disease (binary), tumour height (continuous) |
| 2 | Age (continuous), sex (binary), ASA grade (binary), cT (4 categories), cN (3 categories), neoadjuvant therapy (binary), BMI (binary), diabetes (binary), cardiovascular disease (binary), tumour height (continuous) |
| 3 | Age (continuous), sex (binary), ASA grade (binary), neoadjuvant therapy (binary), BMI (binary), diabetes (binary), cardiovascular disease (binary), tumour height (continuous) |
| 4 | Sex (binary), neoadjuvant therapy (binary), BMI (binary), diabetes (binary), cardiovascular disease (binary), tumour height (continuous) |
| 5 | Sex (binary), neoadjuvant therapy (binary), BMI (binary), diabetes (binary), cardiovascular disease (binary), tumour height (3 categories) |
| 6 | Sex (binary), neoadjuvant therapy (binary), BMI (binary), diabetes (binary), tumour height (continuous) |
| 7 | Sex (binary), neoadjuvant therapy (binary), BMI (binary), diabetes (binary), tumour height (3 categories) |

**Supplementary Table 4.** Predictor overview for models 1–4.

| Predictors | Model 1 | | Model 2 | | Model 3 | | Model 4 | |
| --- | --- | --- | --- | --- | --- | --- | --- | --- |
|  | **OR (95% CI)** | **P** | **OR (95% CI)** | **P** | **OR (95% CI)** | **P** | **OR (95% CI)** | **P** |
| Age (years) | 0.99 (0.98, 1.01) | 0.483 | 0.99 (0.98, 1.01) | 0.463 | 1.00 (0.98, 1.01) | 0.575 |  |  |
| Male sex | 1.91 (1.38, 2.63) | <0.001 | 1.99 (1.44, 2.74) | <0.001 | 1.99 (1.44, 2.74) | <0.001 | 2.01 (1.46, 2.77) | <0.001 |
| BMI (continuous) | 1.07 (1.03, 1.12) | 0.001 |  |  |  |  |  |  |
| BMI (>30 kg/m^2^) |  |  | 1.82 (1.20, 2.74) | 0.004 | 1.84 (1.22, 2.78) | 0.004 | 1.82 (1.21, 2.74) | 0.004 |
| ASA grade II | 0.77 (0.55, 1.07) | 0.121 | 0.78 (0.56, 1.10) | 0.153 | 0.77 (0.55, 1.08) | 0.135 |  |  |
| Diabetes | 1.45 (0.87, 2.39) | 0.150 | 1.47 (0.89, 2.43) | 0.131 | 1.48 (0.90, 2.44) | 0.126 | 1.35 (0.83, 2.20) | 0.232 |
| Cardiovascular disease | 1.35 (0.88, 2.07) | 0.172 | 1.38 (0.90, 2.11) | 0.142 | 1.38 (0.90, 2.12) | 0.135 | 1.27 (0.84, 1.92) | 0.257 |
| Clinical T stage |  |  |  |  |  |  |  |  |
| cT3 | 1.38 (0.92, 2.07) | 0.125 | 1.37 (0.91, 2.06) | 0.131 |  |  |  |  |
| cT4 | 0.78 (0.35, 1.73) | 0.540 | 0.74 (0.34, 1.62) | 0.450 |  |  |  |  |
| cTX | 1.37 (0.53, 3.52) | 0.517 | 1.31 (0.51, 3.36) | 0.580 |  |  |  |  |
| Clinical N stage |  |  |  |  |  |  |  |  |
| cN1–2 | 0.86 (0.61, 1.23) | 0.406 | 0.85 (0.60, 1.20) | 0.362 |  |  |  |  |
| cNX | 0.78 (0.33, 1.85) | 0.570 | 0.77 (0.33, 1.84) | 0.561 |  |  |  |  |
| Neoadjuvant radiotherapy |  |  |  |  |  |  |  |  |
| Radiotherapy | 1.88 (1.23, 2.88) | 0.003 |  |  |  |  |  |  |
| Chemoradiotherapy | 1.63 (0.94, 2.82) | 0.082 |  |  |  |  |  |  |
| Any radiotherapy |  |  | 1.81 (1.18, 2.76) | 0.006 | 1.85 (1.31, 2.63) | 0.001 | 1.89 (1.34, 2.67) | <0.001 |
| Tumour height (cm) | 0.94 (0.87, 1.01) | 0.086 | 0.94 (0.87, 1.01) | 0.093 | 0.94 (0.87, 1.01) | 0.074 | 0.93 (0.87, 1.00) | 0.065 |

**Supplementary Table 5.** Predictor overview for models 5–7.

| Predictors | Model 5 | | Model 6 | | Model 7 |  |
| --- | --- | --- | --- | --- | --- | --- |
|  | **OR (95% CI)** | **P** | **OR (95% CI)** | **P** | **OR (95% CI)** | **P** |
| Male sex | 2.00 (1.45, 2.75) | <0.001 | 2.05 (1.49, 2.82) | <0.001 | 2.04 (1.48, 2.80) | <0.001 |
| BMI (>30 kg/m2) | 1.82 (1.21, 2.74) | 0.004 | 1.81 (1.20, 2.72) | 0.004 | 1.81 (1.20, 2.72) | 0.005 |
| Diabetes | 1.35 (0.83, 2.20) | 0.231 | 1.38 (0.85, 2.24) | 0.195 | 1.38 (0.85, 2.24) | 0.195 |
| Cardiovascular disease | 1.27 (0.84, 1.91) | 0.261 |  |  |  |  |
| Any radiotherapy | 1.90 (1.35, 2.69) | <0.001 | 1.87 (1.32, 2.65) | <0.001 | 1.89 (1.34, 2.67) | <0.001 |
| Tumour height (cm) |  |  | 0.93 (0.87, 1.00) | 0.067 |  |  |
| Tumour height |  |  |  |  |  |  |
| 9 to 10 cm | 0.81 (0.58, 1.12) | 0.201 |  |  | 0.80 (0.58, 1.12) | 0.199 |
| 11 to 12 cm | 0.75 (0.51, 1.10) | 0.144 |  |  | 0.75 (0.51, 1.11) | 0.151 |

**Supplementary Table 6.** Model performance overview.

|  | Model 1 | Model 2 | Model 3 | Model 4 | Model 5 | Model 6 | Model 7 |
| --- | --- | --- | --- | --- | --- | --- | --- |
| Original train AUC (95% CI) | 0.663  (0.626, 0.701) | 0.659  (0.620, 0.698) | 0.652  (0.613, 0.690) | 0.644  (0.605, 0.683) | 0.644  (0.606, 0.683) | 0.641  (0.603, 0.680) | 0.643  (0.605, 0.681) |
| Bootstrap test AUC (95% CI) | 0.649  (0.634, 0.659) | 0.645  (0.629, 0.656) | 0.643  (0.630, 0.652) | 0.638  (0.623, 0.645) | 0.636  (0.621, 0.645) | 0.637  (0.619, 0.643) | 0.635  (0.620, 0.644) |
| Optimism corrected AUC | 0.633  (0.596, 0.671) | 0.631  (0.593, 0.670) | 0.635  (0.596, 0.673) | 0.632  (0.593, 0.671) | 0.629  (0.590, 0.668) | 0.632  (0.593, 0.671) | 0.631  (0.593, 0.669) |
| Original train calibration slope (95% CI) | 0.906  (0.674, 1.137) | 0.900  (0.663, 1.138) | 0.899  (0.649, 1.149) | 0.907  (0.647, 1.168) | 0.907  (0.645, 1.169) | 0.908  (0.645, 1.172) | 0.908  (0.642, 1.173) |
| Bootstrap test  calibration slope (95% CI) | 0.721  (0.552, 0.898) | 0.728  (0.557, 0.923) | 0.791  (0.606, 1.034) | 0.827  (0.634, 1.078) | 0.809  (0.621, 1.049) | 0.848  (0.647, 1.142) | 0.828  (0.631, 1.085) |
| Original train calibration intercept (95% CI) | -0.114  (-0.260, 0.033) | -0.112  (-0.259, 0.034) | -0.109  (-0.255, 0.037) | -0.107  (-0.253, 0.039) | -0.106  (-0.252, 0.040) | -0.106  (-0.252, 0.040) | -0.106  (-0.252, 0.040) |
| Bootstrap test  calibration intercept (95% CI) | -0.123  (-0.283, 0.052) | -0.119  (-0.283, 0.051) | -0.113  (-0.273, 0.056) | -0.108  (-0.267, 0.058) | -0.109  (-0.267, 0.059) | -0.107  (-0.264, 0.059) | -0.107  (-0.267, 0.059) |
| Average MAPE | 0.018 | 0.017 | 0.014 | 0.012 | 0.013 | 0.011 | 0.012 |

**Original train AUC** = Performance based on original data (predicting outcome in original data)

**Bootstrap test AUC** = Performance based on 500 bootstrap samples (predicting outcome in original data)

**Optimism** = average difference between the bootstrap training performance and the bootstrap test performance.

**Optimism corrected AUC** = Original train AUC - Optimism

**Supplementary Table 7.** Classification results evaluated on original data (95% bootstrap confidence intervals), using leakage threshold of 0.10.

|  | Model 1 | Model 2 | Model 3 | Model 4 | Model 5 | Model 6 | Model 7 |
| --- | --- | --- | --- | --- | --- | --- | --- |
| Sensitivity | 48.2  (37.3, 61.9) | 47.7  (36.5, 62.9) | 49.2  (30.2, 62.4) | 51.3  (23.5, 64.7) | 50.8  (24.8, 64.6) | 50.8  (21.0, 66.8) | 49.7  (24.8, 65.3) |
| Specificity | 73.3  (65.9, 81.1) | 73.5  (65.3, 82.3) | 72.6  (62.8, 84.0) | 67.7  (59.4, 88.2) | 67.8  (59.6, 87.9) | 68.8  (59.4, 88.9) | 69.3  (59.2, 89.6) |
| Negative predictive value | 94.7  (94.2, 95.7) | 94.7  (94.2, 95.8) | 94.8  (93.9, 95.6) | 94.6  (93.7, 95.6) | 94.6  (93.7, 95.6) | 94.7  (93.7, 95.8) | 94.6  (93.6, 95.7) |
| Positive predictive value | 12.5  (11.3, 14.7) | 12.4  (11.2, 14.8) | 12.5  (10.7, 14.6) | 11.1  (10.1, 13.9) | 11.1  (10.1, 14.3) | 11.3  (9.6, 13.7) | 11.3  (10.0, 14.1) |

**Supplementary Table 8.** Simple scoring systems for probability of anastomotic leakage after low anterior resection for rectal cancer based on the derived models described in Supplementary Table 4 and Supplementary Table 5.

|  | Points | | | | | | |
| --- | --- | --- | --- | --- | --- | --- | --- |
|  | **Model 1** | **Model 2** | **Model 3** | **Model 4** | **Model 5** | **Model 6** | **Model 7** |
| Age |  |  |  |  |  |  |  |
| ≤60 years | 0 | 0 | 0 |  |  |  |  |
| 61–69 years | 0 | 0 | 0 |  |  |  |  |
| ≥70 years | 0 | 0 | 0 |  |  |  |  |
| Sex |  |  |  |  |  |  |  |
| Male | 1 | 1 | 1 | 1 | 1 | 1 | 1 |
| Female | 0 | 0 | 0 | 0 | 0 | 0 | 0 |
| BMI (continuous) |  |  |  |  |  |  |  |
| <25 kg/m^2^ | 0 |  |  |  |  |  |  |
| 25–30 kg/m^2^ | 1 |  |  |  |  |  |  |
| ≥30 kg/m^2^ | 2 |  |  |  |  |  |  |
| BMI (binary) |  |  |  |  |  |  |  |
| <30 kg/m^2^ |  | 0 | 0 | 0 | 0 | 0 | 0 |
| ≥30 kg/m^2^ |  | 1 | 1 | 1 | 1 | 1 | 1 |
| ASA grade |  |  |  |  |  |  |  |
| I | 0 | 0 | 0 |  |  |  |  |
| II | 0 | 0 | 0 |  |  |  |  |
| Diabetes |  |  |  |  |  |  |  |
| No | 0 | 0 | 0 | 0 | 0 | 0 | 0 |
| Yes | 1 | 1 | 1 | 0 | 0 | 0 | 0 |
| Cardiovascular disease |  |  |  |  |  |  |  |
| No | 0 | 0 | 0 | 0 | 0 |  |  |
| Yes | 0 | 0 | 0 | 0 | 0 |  |  |
| Clinical T stage |  |  |  |  |  |  |  |
| cT1–2 | 0 | 0 |  |  |  |  |  |
| cT3 | 0 | 0 |  |  |  |  |  |
| cT4 | 0 | 0 |  |  |  |  |  |
| cTX | 0 | 0 |  |  |  |  |  |
| Clinical N stage |  |  |  |  |  |  |  |
| cN0 | 0 | 0 |  |  |  |  |  |
| cN1–2 | 0 | 0 |  |  |  |  |  |
| cNX | 0 | 0 |  |  |  |  |  |
| Neoadjuvant radiotherapy |  |  |  |  |  |  |  |
| No | 0 |  |  |  |  |  |  |
| Radiotherapy | 1 |  |  |  |  |  |  |
| Chemoradiotherapy | 1 |  |  |  |  |  |  |
| Any radiotherapy (binary) |  |  |  |  |  |  |  |
| No |  | 0 | 0 | 0 | 0 | 0 | 0 |
| Yes |  | 1 | 1 | 1 | 1 | 1 | 1 |
| Tumour height (continuous) |  |  |  |  |  |  |  |
| ≤8 cm | 1 | 1 | 1 | 1 |  | 1 |  |
| 9–10 cm | 0 | 0 | 0 | 0 |  | 0 |  |
| 11–12 cm | 0 | 0 | 0 | 0 |  | 0 |  |
| Tumour height (categorical) |  |  |  |  |  |  |  |
| 0–8 cm |  |  |  |  | 0 |  | 0 |
| 9–10 cm |  |  |  |  | 0 |  | 0 |
| 11–12 cm |  |  |  |  | 0 |  | 0 |

**Supplementary Table 9.** Scores according to the scoring systems in Supplementary Table 8 and corresponding predicted risks of anastomotic leakage according to the developed models in Supplementary Table 4 and Supplementary Table 5. Scores and risk estimates are computed for one imputed data set.

| Score | Patients (n) | Predicted leak risk (%) | | | | | Patients (n) with predicted leak risk > 10% |
| --- | --- | --- | --- | --- | --- | --- | --- |
|  |  | Min | Q1 | Median | Q3 | Max |  |
| *Model 1* |  |  |  |  |  |  |  |
| 0 | 168 | 1.2 | 1.8 | 2.2 | 2.7 | 5.1 | 0 |
| 1 | 508 | 1.3 | 3.0 | 3.9 | 5.0 | 8.5 | 0 |
| 2 | 937 | 2.0 | 4.9 | 6.2 | 8.1 | 16.1 | 108 |
| 3 | 743 | 3.3 | 7.4 | 9.7 | 12.4 | 22.7 | 331 |
| 4 | 311 | 5.1 | 11.5 | 14.3 | 17.3 | 28.4 | 272 |
| 5 | 56 | 12.0 | 17.7 | 20.4 | 23.9 | 37.2 | 56 |
| 6 | 4 | 19.5 | 21.0 | 22.4 | 26.5 | 36.0 | 4 |
| *Model 2* |  |  |  |  |  |  |  |
| 0 | 247 | 1.2 | 2.2 | 2.5 | 3.0 | 5.3 | 0 |
| 1 | 820 | 2.1 | 3.9 | 4.8 | 5.8 | 10.5 | 1 |
| 2 | 1089 | 2.6 | 6.2 | 8.1 | 10.3 | 18.0 | 292 |
| 3 | 498 | 4.7 | 10.6 | 13.3 | 15.8 | 33.1 | 401 |
| 4 | 69 | 9.1 | 16.9 | 20.5 | 24.5 | 35.0 | 68 |
| 5 | 4 | 20.8 | 25.7 | 29.3 | 32.9 | 37.9 | 4 |
| *Model 3* |  |  |  |  |  |  |  |
| 0 | 247 | 2.0 | 2.3 | 2.5 | 3.0 | 4.5 | 0 |
| 1 | 820 | 2.6 | 4.2 | 4.7 | 5.6 | 9.0 | 0 |
| 2 | 1089 | 3.8 | 6.5 | 8.3 | 10.2 | 16.8 | 284 |
| 3 | 498 | 7.2 | 10.6 | 13.0 | 14.8 | 27.3 | 434 |
| 4 | 69 | 13.5 | 16.8 | 20.7 | 24.4 | 43.0 | 69 |
| 5 | 4 | 26.8 | 27.4 | 28.9 | 31.8 | 36.7 | 4 |
| *Model 4* |  |  |  |  |  |  |  |
| 0 | 260 | 2.3 | 2.5 | 2.6 | 2.8 | 4.5 | 0 |
| 1 | 862 | 3.0 | 4.6 | 5.0 | 5.3 | 9.1 | 0 |
| 2 | 1118 | 5.5 | 6.5 | 8.7 | 10.0 | 18.3 | 433 |
| 3 | 456 | 9.7 | 11.5 | 12.3 | 15.5 | 27.2 | 455 |
| 4 | 31 | 16.3 | 20.9 | 22.4 | 26.1 | 32.6 | 31 |
| *Model 5* |  |  |  |  |  |  |  |
| 0 | 377 | 2.5 | 2.5 | 2.6 | 3.3 | 5.6 | 0 |
| 1 | 1204 | 3.5 | 5.0 | 5.3 | 6.6 | 11.1 | 2 |
| 2 | 1061 | 5.6 | 10.0 | 11.3 | 12.4 | 21.3 | 827 |
| 3 | 85 | 11.3 | 17.7 | 18.2 | 22.6 | 30.5 | 85 |
| *Model 6* |  |  |  |  |  |  |  |
| 0 | 260 | 2.4 | 2.5 | 2.7 | 2.7 | 4.0 | 0 |
| 1 | 862 | 3.1 | 4.7 | 5.1 | 5.4 | 8.2 | 0 |
| 2 | 1118 | 5.6 | 6.6 | 9.1 | 10.4 | 15.4 | 405 |
| 3 | 456 | 8.0 | 11.9 | 12.7 | 14.6 | 27.8 | 455 |
| 4 | 31 | 16.9 | 21.5 | 23.0 | 24.6 | 31.8 | 31 |
| *Model 7* |  |  |  |  |  |  |  |
| 0 | 377 | 2.5 | 2.5 | 2.7 | 3.3 | 4.6 | 0 |
| 1 | 1204 | 3.6 | 5.1 | 5.5 | 6.3 | 9.4 | 0 |
| 2 | 1061 | 5.7 | 9.9 | 10.4 | 12.9 | 22.5 | 789 |
| 3 | 85 | 11.6 | 17.6 | 18.7 | 23.3 | 32.1 | 85 |

**Supplementary Table 10.** Scoring system derivation based on Model 1. *B* = b_2_ = 0.6451.

| Predictor | Categories | Reference  value (*W_ij_*) | b*_i_* | *D_ij_* =  b*_i_* (*W_ij_-W_i_*_REF_) | Rescaled *D_ij_* | Points*_ij_* =  (Rescaled *D_ij_)/B* |
| --- | --- | --- | --- | --- | --- | --- |
| Age |  |  | -0.0056 |  |  |  |
|  | ≤60 years | 41.5 = *W_1_*_REF_ |  | 0 | 0.1877 | 0 |
|  | 61–69 years | 65.0 |  | -0.1317 | 0.0560 | 0 |
|  | ≥70 years | 75.0 |  | -0.1877 | 0 | 0 |
| Sex |  |  | 0.6451 |  |  |  |
|  | Female | 0 = *W_2_*_REF_ |  | 0 | 0 | 0 |
|  | Male | 1 |  | 0.6451 | 0.6451 | 1 |
| BMI (continuous) |  |  | 0.0695 |  |  |  |
|  | <25 kg/m^2^ | 19.5 = *W_3_*_REF_ |  | 0 | 0 | 0 |
|  | 25–30 kg/m^2^ | 27.0 |  | 0.5210 | 0.5210 | 1 |
|  | ≥30 kg/m^2^ | 40.0 |  | 1.4240 | 1.4240 | 2 |
| ASA grade |  |  | -0.2677 |  |  |  |
|  | I | 0 = *W_4_*_REF_ |  | 0 | 0.2677 | 0 |
|  | II | 1 |  | -0.2677 | 0 | 0 |
| Diabetes |  |  | 0.3690 |  |  |  |
|  | No | 0 = *W_5_*_REF_ |  | 0 | 0 | 0 |
|  | Yes | 1 |  | 0.3690 | 0.3690 | 1 |
| Cardiovascular disease |  |  | 0.2979 |  |  |  |
|  | No | 0 = *W_6_*_REF_ |  | 0 | 0 | 0 |
|  | Yes | 1 |  | 0.2979 | 0.2979 | 0 |
| Clinical T stage |  |  |  |  |  |  |
| cT3 |  |  | 0.3190 |  |  |  |
|  | No | 0 = *W_7_*_REF_ |  | 0 | 0 | 0 |
|  | Yes | 1 |  | 0.3190 | 0.3190 | 0 |
| cT4 |  |  | -0.2500 |  |  |  |
|  | No | 0 = *W_8_*_REF_ |  | 0 | 0.2500 | 0 |
|  | Yes | 1 |  | -0.2500 | 0 | 0 |
| cTX |  |  | 0.3128 |  |  |  |
|  | No | 0 = *W_9_*_REF_ |  | 0 | 0 | 0 |
|  | Yes | 1 |  | 0.3128 | 0.3128 | 0 |
| Clinical N stage |  |  |  |  |  |  |
| cN1–2 |  |  | -0.1493 |  |  |  |
|  | No | 0 = *W_10_*_REF_ |  | 0 | 0.1493 | 0 |
|  | Yes | 1 |  | -0.1493 | 0 | 0 |
| cNX |  |  | -0.2505 |  |  |  |
|  | No | 0 = *W_11_*_REF_ |  | 0 | 0.2505 | 0 |
|  | Yes | 1 |  | -0.2505 | 0 | 0 |
| Neoadjuvant radiotherapy |  |  |  |  |  |  |
| Radiotherapy |  |  | 0.6334 |  |  |  |
|  | No | 0 = *W_12_*_REF_ |  | 0 | 0 | 0 |
|  | Yes | 1 |  | 0.6334 | 0.6334 | 1 |
| Chemoradiotherapy |  |  | 0.4879 |  |  |  |
|  | No | 0 = *W_13_*_REF_ |  | 0 | 0 | 0 |
|  | Yes | 1 |  | 0.4879 | 0.4879 | 1 |
| Tumour height (continuous) |  |  | -0.0647 |  |  |  |
|  | ≤8 cm | 4.5 = *W_14_*_REF_ |  | 0 | 0.4427 | 1 |
|  | 9–10 cm | 9.5 |  | -0.3233 | 0.1293 | 0 |
|  | 11–12 cm | 11.5 |  | -0.4527 | 0 | 0 |

**Supplementary Table 11.** Scoring system derivation based on Model 2. *B* = b_2_ = 0.6859.

| Predictor | Categories | Reference  value (*W_ij_*) | b*_i_* | *D_ij_* =  b*_i_* (*W_ij_-W_i_*_REF_) | Rescaled *D_ij_* | Points*_ij_* =  (Rescaled *D_ij_)/B* |
| --- | --- | --- | --- | --- | --- | --- |
| Age |  |  | -0.0058 |  |  |  |
|  | ≤60 years | 41.5 = *W_1_*_REF_ |  | 0 | 0.1931 | 0 |
|  | 61–69 years | 65.0 |  | -0.1355 | 0.0576 | 0 |
|  | ≥70 years | 75.0 |  | -0.1931 | 0 | 0 |
| Sex |  |  | 0.6859 |  |  |  |
|  | Female | 0 = *W_2_*_REF_ |  | 0 | 0 | 0 |
|  | Male | 1 |  | 0.6859 | 0.6859 | 1 |
| BMI (binary) |  |  | 0.5975 |  |  |  |
|  | <30 kg/m^2^ | 0 = *W_3_*_REF_ |  | 0 | 0 | 0 |
|  | ≥30 kg/m^2^ | 1 |  | 0.5975 | 0.5975 | 1 |
| ASA grade |  |  | -0.2456 |  |  |  |
|  | I | 0 = *W_4_*_REF_ |  | 0 | 0.2456 | 0 |
|  | II | 1 |  | -0.2456 | 0 | 0 |
| Diabetes |  |  | 0.3868 |  |  |  |
|  | No | 0 = *W_5_*_REF_ |  | 0 | 0 | 0 |
|  | Yes | 1 |  | 0.3868 | 0.3868 | 1 |
| Cardiovascular disease |  |  | 0.3199 |  |  |  |
|  | No | 0 = *W_6_*_REF_ |  | 0 | 0 | 0 |
|  | Yes | 1 |  | 0.3199 | 0.3199 | 0 |
| Clinical T stage |  |  |  |  |  |  |
| cT3 |  |  | 0.3140 |  |  |  |
|  | No | 0 = *W_8_*_REF_ |  | 0 | 0 | 0 |
|  | Yes | 1 |  | 0.3140 | 0.3140 | 0 |
| cT4 |  |  | -0.3006 |  |  |  |
|  | No | 0 = *W_9_*_REF_ |  | 0 | 0.3006 | 0 |
|  | Yes | 1 |  | -0.3006 | 0 | 0 |
| cTX |  |  | 0.2669 |  |  |  |
|  | No | 0 = *W_10_*_REF_ |  | 0 | 0 | 0 |
|  | Yes | 1 |  | 0.2669 | 0.2669 | 0 |
| Clinical N stage |  |  |  |  |  |  |
| cN1–2 |  |  | -0.1619 |  |  |  |
|  | No | 0 = *W_11_*_REF_ |  | 0 | 0.1619 | 0 |
|  | Yes | 1 |  | -0.1619 | 0 | 0 |
| cNX |  |  | -0.2562 |  |  |  |
|  | No | 0 = *W_12_*_REF_ |  | 0 | 0.2562 | 0 |
|  | Yes | 1 |  | -0.2562 | 0 | 0 |
| Any radiotherapy (binary) |  |  | 0.5924 |  |  |  |
|  | No | 0 = *W_13_*_REF_ |  | 0 | 0 | 0 |
|  | Yes | 1 |  | 0.5924 | 0.5924 | 1 |
| Tumour height (continuous) |  |  | -0.0631 |  |  |  |
|  | ≤8 cm | 4.5 = *W_14_*_REF_ |  | 0 | 0.4420 | 1 |
|  | 9–10 cm | 9.5 |  | -0.3157 | 0.1263 | 0 |
|  | 11–12 cm | 11.5 |  | -0.4420 | 0 | 0 |

**Supplementary Table 12.** Scoring system derivation based on Model 3. *B* = b_2_ = 0.6878.

| Predictor | Categories | Reference  value (*W_ij_*) | b*_i_* | *D_ij_* =  b*_i_* (*W_ij_-W_i_*_REF_) | Rescaled *D_ij_* | Points*_ij_* =  (Rescaled *D_ij_)/B* |
| --- | --- | --- | --- | --- | --- | --- |
| Age |  |  | -0.0044 |  |  |  |
|  | ≤60 years | 41.5 = *W_1_*_REF_ |  | 0 | 0.1458 | 0 |
|  | 61–69 years | 65.0 |  | -0.1023 | 0.0435 | 0 |
|  | ≥70 years | 75.0 |  | -0.1458 | 0 | 0 |
| Sex |  |  | 0.6878 |  |  |  |
|  | Female | 0 = *W_2_*_REF_ |  | 0 | 0 | 0 |
|  | Male | 1 |  | 0. 6878 | 0. 6878 | 1 |
| BMI (binary) |  |  | 0.6105 |  |  |  |
|  | <30 kg/m^2^ | 0 = *W_3_*_REF_ |  | 0 | 0 | 0 |
|  | ≥30 kg/m^2^ | 1 |  | 0. 6105 | 0. 6105 | 1 |
| ASA grade |  |  | -0.2550 |  |  |  |
|  | I | 0 = *W_4_*_REF_ |  | 0 | 0. 2550 | 0 |
|  | II | 1 |  | -0. 2550 | 0 | 0 |
| Diabetes |  |  | 0.3910 |  |  |  |
|  | No | 0 = *W_5_*_REF_ |  | 0 | 0 | 0 |
|  | Yes | 1 |  | 0. 3910 | 0. 3910 | 1 |
| Cardiovascular disease |  |  | 0.3242 |  |  |  |
|  | No | 0 = *W_6_*_REF_ |  | 0 | 0 | 0 |
|  | Yes | 1 |  | 0. 3242 | 0. 3242 | 0 |
| Any radiotherapy (binary) |  |  | 0.6179 |  |  |  |
|  | No | 0 = *W_7_*_REF_ |  | 0 | 0 | 0 |
|  | Yes | 1 |  | 0. 6179 | 0. 6179 | 1 |
| Tumour height (continuous) |  |  | -0.0663 |  |  |  |
|  | ≤8 cm | 4.5 = *W_8_*_REF_ |  | 0 | 0.4640 | 1 |
|  | 9–10 cm | 9.5 |  | -0.3314 | 0.1326 | 0 |
|  | 11–12 cm | 11.5 |  | -0.4640 | 0 | 0 |

**Supplementary Table 13.** Scoring system derivation based on Model 4. *B* = b_1_ = 0.6963.

| Predictor | Categories | Reference  value (*W_ij_*) | b*_i_* | *D_ij_* =  b*_i_* (*W_ij_-W_i_*_REF_) | Rescaled *D_ij_* | Points*_ij_* =  (Rescaled *D_ij_)/B* |
| --- | --- | --- | --- | --- | --- | --- |
| Sex |  |  | 0.6963 |  |  |  |
|  | Female | 0 = *W_1_*_REF_ |  | 0 | 0 | 0 |
|  | Male | 1 |  | 0. 6963 | 0. 6963 | 1 |
| BMI (binary) |  |  | 0.6005 |  |  |  |
|  | <30 kg/m^2^ | 0 = *W_2_*_REF_ |  | 0 | 0 | 0 |
|  | ≥30 kg/m^2^ | 1 |  | 0. 6005 | 0. 6005 | 1 |
| Diabetes |  |  | 0.2980 |  |  |  |
|  | No | 0 = *W_3_*_REF_ |  | 0 | 0 | 0 |
|  | Yes | 1 |  | 0. 2980 | 0. 2980 | 0 |
| Cardiovascular disease |  |  | 0.2381 |  |  |  |
|  | No | 0 = *W_4_*_REF_ |  | 0 | 0 | 0 |
|  | Yes | 1 |  | 0. 2381 | 0. 2381 | 0 |
| Neoadjuvant therapy (binary) |  |  | 0.6360 |  |  |  |
|  | No | 0 = *W_5_*_REF_ |  | 0 | 0 | 0 |
|  | Yes | 1 |  | 0. 6360 | 0. 6360 | 1 |
| Tumour height (continuous) |  |  | -0.0683 |  |  |  |
|  | ≤8 cm | 4.5 = *W_6_*_REF_ |  | 0 | 0.4782 | 1 |
|  | 9–10 cm | 9.5 |  | -0.3415 | 0.1366 | 0 |
|  | 11–12 cm | 11.5 |  | -0.4782 | 0 | 0 |

**Supplementary Table 14.** Scoring system derivation based on Model 5. *B* = b_1_ = 0.6916.

| Predictor | Categories | Reference  value (*W_ij_*) | b*_i_* | *D_ij_* =  b*_i_* (*W_ij_-W_i_*_REF_) | Rescaled *D_ij_* | Points*_ij_* =  (Rescaled *D_ij_)/B* |
| --- | --- | --- | --- | --- | --- | --- |
| Sex |  |  | 0.6916 |  |  |  |
|  | Female | 0 = *W_1_*_REF_ |  | 0 | 0 | 0 |
|  | Male | 1 |  | 0. 6916 | 0. 6916 | 1 |
| BMI (binary) |  |  | 0.5999 |  |  |  |
|  | <30 kg/m^2^ | 0 = *W_2_*_REF_ |  | 0 | 0 | 0 |
|  | ≥30 kg/m^2^ | 1 |  | 0. 5999 | 0. 5999 | 1 |
| Diabetes |  |  | 0.2984 |  |  |  |
|  | No | 0 = *W_3_*_REF_ |  | 0 | 0 | 0 |
|  | Yes | 1 |  | 0. 2984 | 0. 2984 | 0 |
| Cardiovascular disease |  |  | 0.2360 |  |  |  |
|  | No | 0 = *W_4_*_REF_ |  | 0 | 0 | 0 |
|  | Yes | 1 |  | 0. 2360 | 0. 2360 | 0 |
| Any radiotherapy (binary) |  |  | 0.6442 |  |  |  |
|  | No | 0 = *W_5_*_REF_ |  | 0 | 0 | 0 |
|  | Yes | 1 |  | 0. 6442 | 0. 6442 | 1 |
| Tumour height (categorical) |  |  |  |  |  |  |
| 9‒10cm |  |  | -0.2166 |  |  |  |
|  | No | 0 = *W_6_*_REF_ |  | 0 | 0. 2166 | 0 |
|  | Yes | 1 |  | -0. 2166 | 0 | 0 |
| 11‒12cm |  |  | -0.2862 |  |  |  |
|  | No | 0 = *W_7_*_REF_ |  | 0 | 0. 2862 | 0 |
|  | Yes | 1 |  | -0. 2862 | 0 | 0 |

**Supplementary Table 15.** Scoring system derivation based on Model 6. *B* = b_1_ = 0.7174.

| Predictor | Categories | Reference  value (*W_ij_*) | b*_i_* | *D_ij_* =  b*_i_* (*W_ij_-W_i_*_REF_) | Rescaled *D_ij_* | Points*_ij_* =  (Rescaled *D_ij_)/B* |
| --- | --- | --- | --- | --- | --- | --- |
| Sex |  |  | 0.7174 |  |  |  |
|  | Female | 0 = *W_1_*_REF_ |  | 0 | 0 | 0 |
|  | Male | 1 |  | 0.7174 | 0. 7174 | 1 |
| BMI (binary) |  |  | 0.5918 |  |  |  |
|  | <30 kg/m^2^ | 0 = *W_2_*_REF_ |  | 0 | 0 | 0 |
|  | ≥30 kg/m^2^ | 1 |  | 0. 5918 | 0. 5918 | 1 |
| Diabetes |  |  | 0.3215 |  |  |  |
|  | No | 0 = *W_3_*_REF_ |  | 0 | 0 | 0 |
|  | Yes | 1 |  | 0. 3215 | 0. 3215 | 0 |
| Any radiotherapy (binary) |  |  | 0.6276 |  |  |  |
|  | No | 0 = *W_4_*_REF_ |  | 0 | 0 | 0 |
|  | Yes | 1 |  | 0. 6276 | 0. 6276 | 1 |
| Tumour height (continuous) |  |  | -0.0676 |  |  |  |
|  | ≤8 cm | 4.5 = *W_5_*_REF_ |  | 0 | 0.4733 | 1 |
|  | 9–10 cm | 9.5 |  | -0.3381 | 0.1352 | 0 |
|  | 11–12 cm | 11.5 |  | -0.4733 | 0 | 0 |

**Supplementary Table 16.** Scoring system derivation based on Model 7. *B* = b_1_ = 0.7125.

| Predictor | Categories | Reference  value (*W_ij_*) | b*_i_* | *D_ij_* =  b*_i_* (*W_ij_-W_i_*_REF_) | Rescaled *D_ij_* | Points*_ij_* =  (Rescaled *D_ij_)/B* |
| --- | --- | --- | --- | --- | --- | --- |
| Sex |  |  | 0.7125 |  |  |  |
|  | Female | 0 = *W_1_*_REF_ |  | 0 | 0 | 0 |
|  | Male | 1 |  | 0. 7125 | 0. 7125 | 1 |
| BMI (binary) |  |  | 0.5910 |  |  |  |
|  | <30 kg/m^2^ | 0 = *W_2_*_REF_ |  | 0 | 0 | 0 |
|  | ≥30 kg/m^2^ | 1 |  | 0. 5910 | 0. 5910 | 1 |
| Diabetes |  |  | 0.3220 |  |  |  |
|  | No | 0 = *W_3_*_REF_ |  | 0 | 0 | 0 |
|  | Yes | 1 |  | 0. 3220 | 0. 3220 | 0 |
| Any radiotherapy (binary) |  |  | 0.6360 |  |  |  |
|  | No | 0 = *W_4_*_REF_ |  | 0 | 0 | 0 |
|  | Yes | 1 |  | 0. 6360 | 0. 6360 | 1 |
| Tumour height (categorical) |  |  |  |  |  |  |
| 9‒10cm |  |  | -0.2179 |  |  |  |
|  | No | 0 = *W_5_*_REF_ |  | 0 | 0.2179 | 0 |
|  | Yes | 1 |  | -0.2179 | 0 | 0 |
| 11‒12cm |  |  | -0.2811 |  |  |  |
|  | No | 0 = *W_6_*_REF_ |  | 0 | 0.2811 | 0 |
|  | Yes | 1 |  | -0.2811 | 0 | 0 |

**
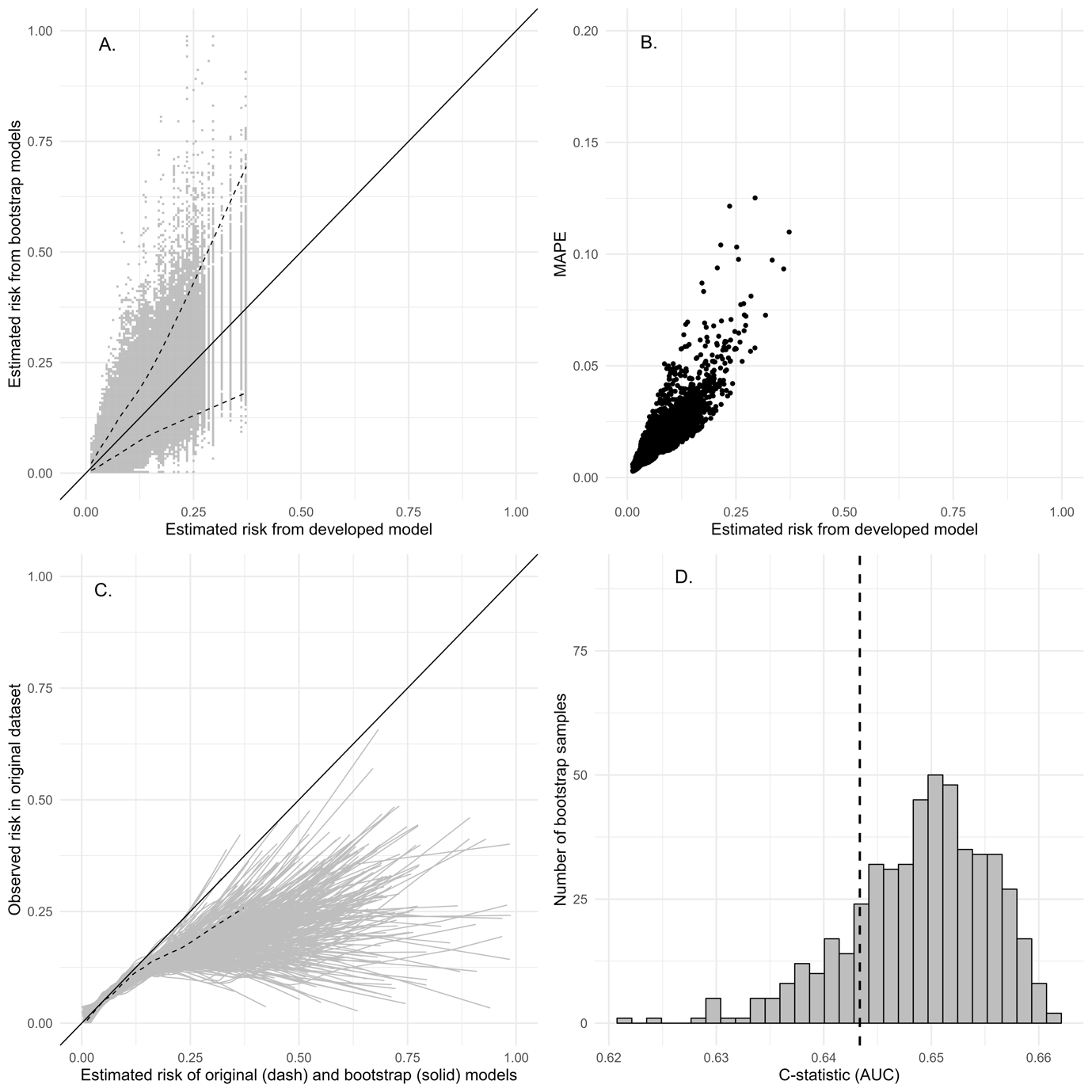
**

**Supplementary Figure 1.** Model 1 instability plots. A. Prediction instability plot. Plot of (pooled) predictions based on original data (x-axis) vs. (pooled) predictions based on bootstrap samples; B. MAPE instability plot. Plot of (pooled) predictions based on original data vs mean absolute prediction error (MAPE) in the bootstrap samples; C. Calibration instability plot. Plot of calibration curves based on bootstrap samples (grey) and original data (black dashed); D. Bootstrap test performance. Histogram of AUCs based on bootstrap samples (5 imputations) used to predict outcome in the original 5 imputed datasets.

**
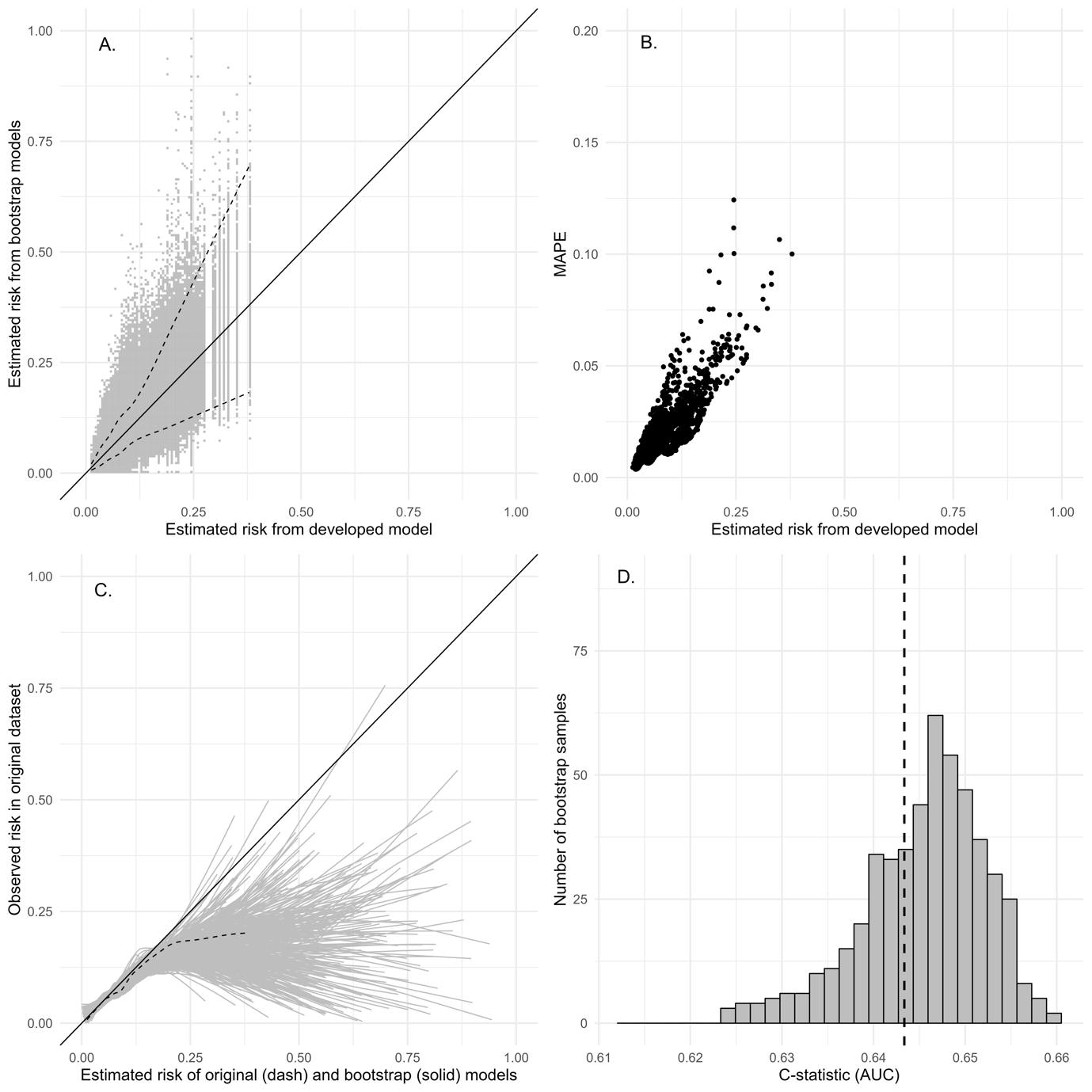
**

**Supplementary Figure 2.** Model 2 instability plots. A. Prediction instability plot. Plot of (pooled) predictions based on original data (x-axis) vs. (pooled) predictions based on bootstrap samples; B. MAPE instability plot. Plot of (pooled) predictions based on original data vs mean absolute prediction error (MAPE) in the bootstrap samples; C. Calibration instability plot. Plot of calibration curves based on bootstrap samples (grey) and original data (black dashed); D. Bootstrap test performance. Histogram of AUCs based on bootstrap samples (5 imputations) used to predict outcome in the original 5 imputed datasets.

**
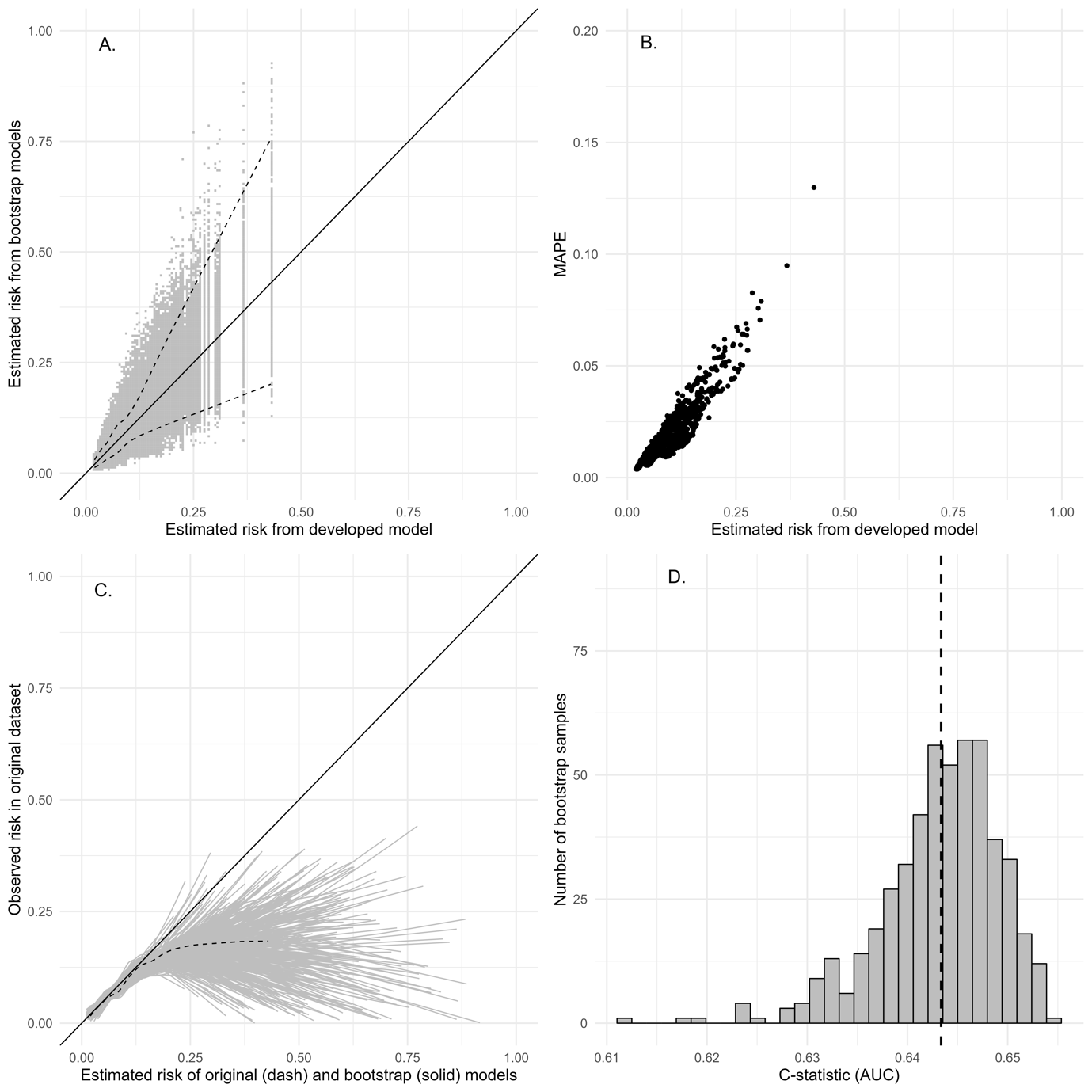
**

**Supplementary Figure 3.** Model 3 instability plots. A. Prediction instability plot. Plot of (pooled) predictions based on original data (x-axis) vs. (pooled) predictions based on bootstrap samples; B. MAPE instability plot. Plot of (pooled) predictions based on original data vs mean absolute prediction error (MAPE) in the bootstrap samples; C. Calibration instability plot. Plot of calibration curves based on bootstrap samples (grey) and original data (black dashed); D. Bootstrap test performance. Histogram of AUCs based on bootstrap samples (5 imputations) used to predict outcome in the original 5 imputed datasets.

**
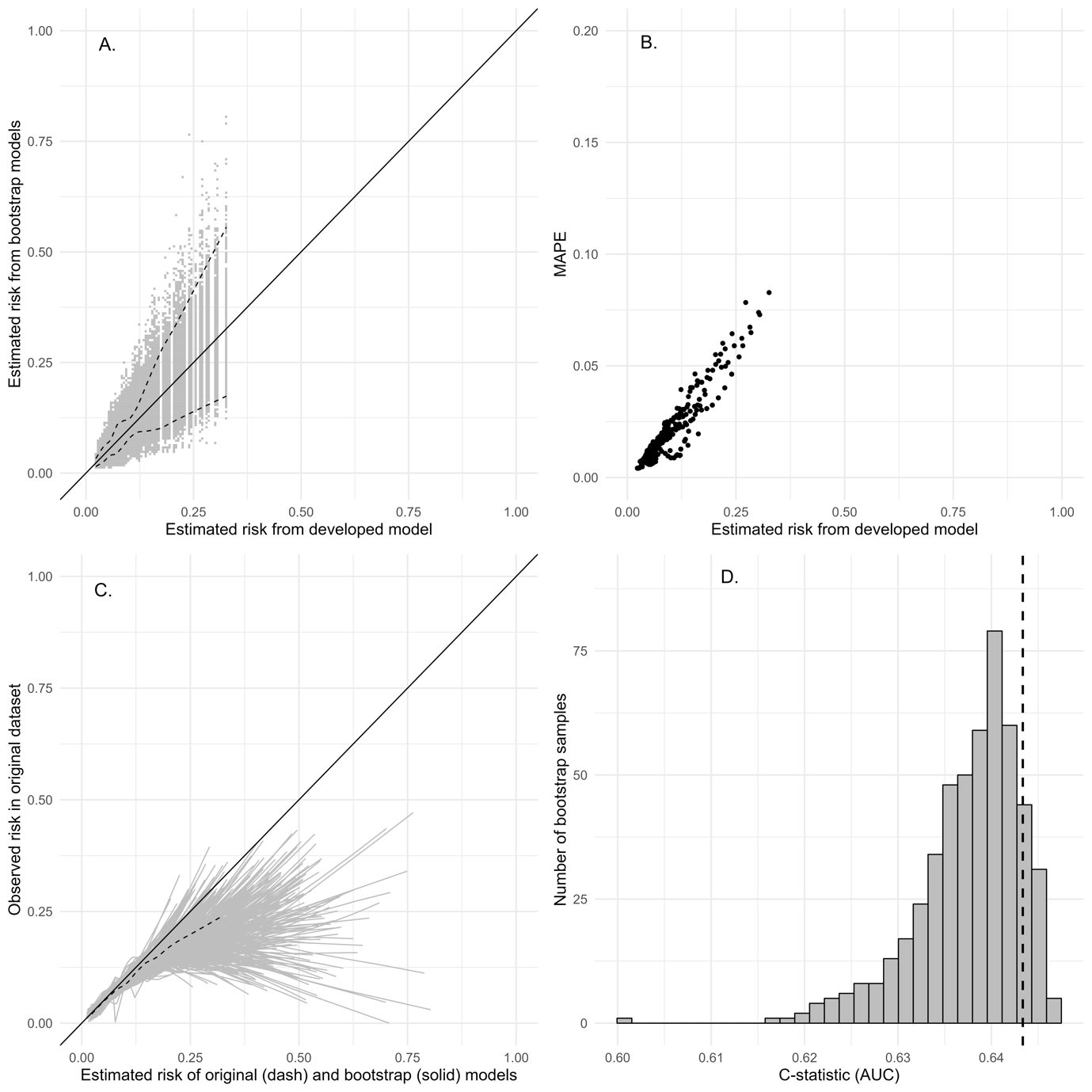
**

**Supplementary Figure 4.** Model 4 instability plots. A. Prediction instability plot. Plot of (pooled) predictions based on original data (x-axis) vs. (pooled) predictions based on bootstrap samples; B. MAPE instability plot. Plot of (pooled) predictions based on original data vs mean absolute prediction error (MAPE) in the bootstrap samples; C. Calibration instability plot. Plot of calibration curves based on bootstrap samples (grey) and original data (black dashed); D. Bootstrap test performance. Histogram of AUCs based on bootstrap samples (5 imputations) used to predict outcome in the original 5 imputed datasets.

**
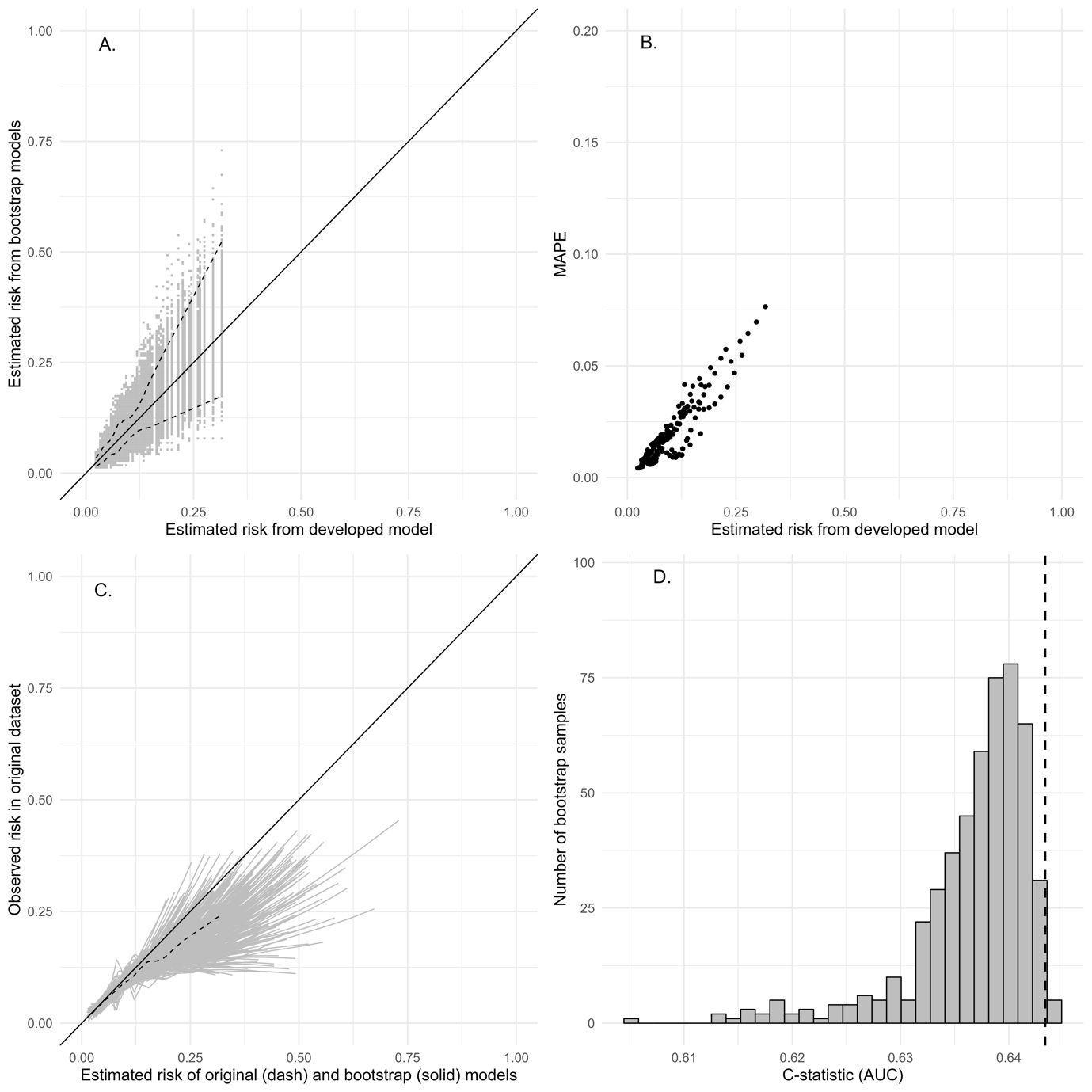
**

**Supplementary Figure 5.** Model 6 instability plots. A. Prediction instability plot. Plot of (pooled) predictions based on original data (x-axis) vs. (pooled) predictions based on bootstrap samples; B. MAPE instability plot. Plot of (pooled) predictions based on original data vs mean absolute prediction error (MAPE) in the bootstrap samples; C. Calibration instability plot. Plot of calibration curves based on bootstrap samples (grey) and original data (black dashed); D. Bootstrap test performance. Histogram of AUCs based on bootstrap samples (5 imputations) used to predict outcome in the original 5 imputed datasets.

**
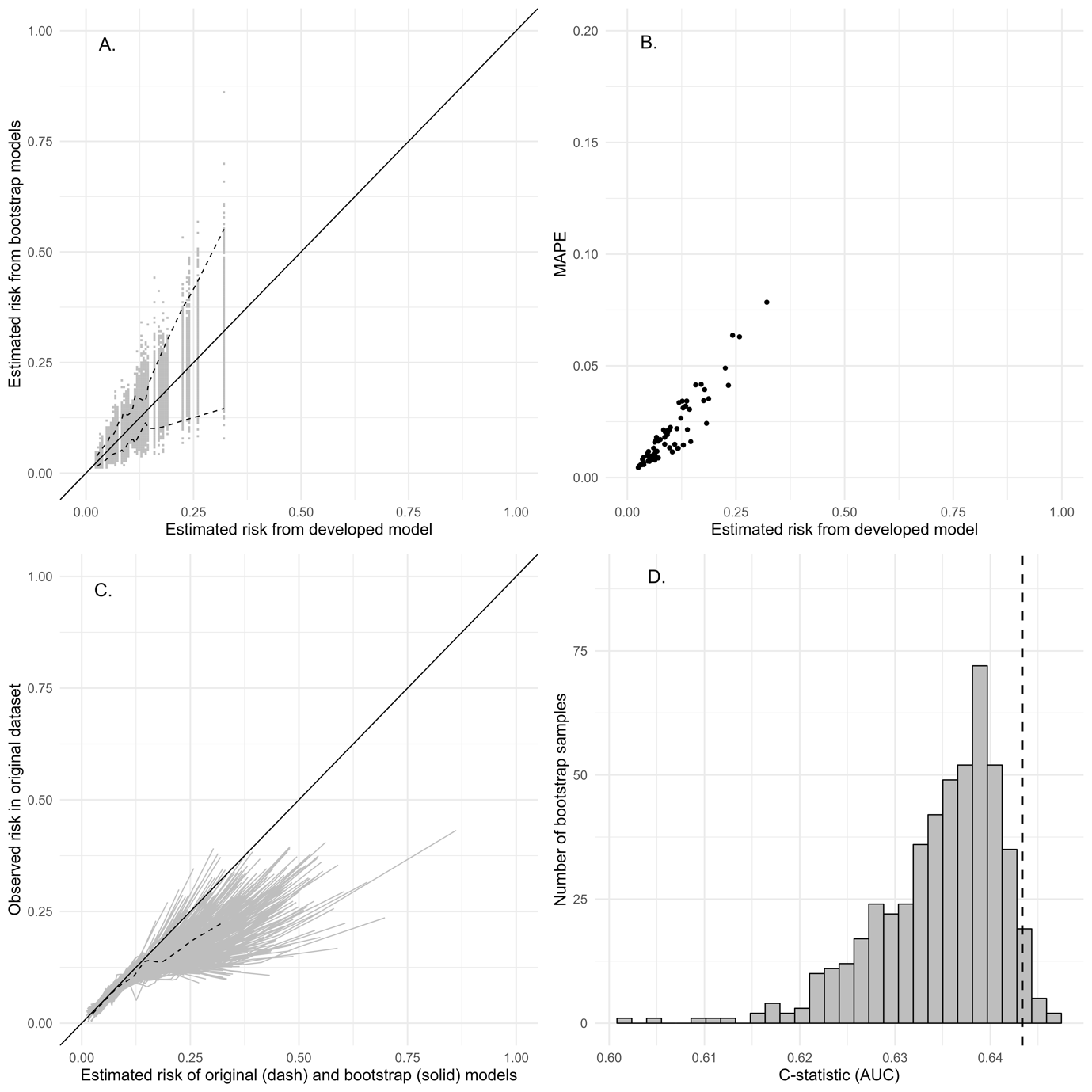
**

**Supplementary Figure 6.** Model 7 instability plots. A. Prediction instability plot. Plot of (pooled) predictions based on original data (x-axis) vs. (pooled) predictions based on bootstrap samples; B. MAPE instability plot. Plot of (pooled) predictions based on original data vs mean absolute prediction error (MAPE) in the bootstrap samples; C. Calibration instability plot. Plot of calibration curves based on bootstrap samples (grey) and original data (black dashed); D. Bootstrap test performance. Histogram of AUCs based on bootstrap samples (5 imputations) used to predict outcome in the original 5 imputed datasets.

d)

c)
